# Potential Use of Untreated Wastewater for Assessing COVID-19 Trends in Southern Italy

**DOI:** 10.3390/ijerph181910278

**Published:** 2021-09-29

**Authors:** Osvalda De Giglio, Francesco Triggiano, Francesca Apollonio, Giusy Diella, Fabrizio Fasano, Pasquale Stefanizzi, Marco Lopuzzo, Silvia Brigida, Carla Calia, Chrysovalentinos Pousis, Angelo Marzella, Giuseppina La Rosa, Luca Lucentini, Elisabetta Suffredini, Giovanna Barbuti, Giuseppina Caggiano, Maria Teresa Montagna

**Affiliations:** 1 Regional Reference Laboratory of SARS-CoV-2 in Wastewater, Department of Biomedical Science and Human Oncology, Hygiene Section, University of Bari Aldo Moro, Piazza G. Cesare 11, 70124 Bari, Italy; mariateresa.montagna@uniba.it; 2 Department of Biomedical Science and Human Oncology, Hygiene Section, University of Bari Aldo Moro, Piazza G. Cesare 11, 70124 Bari, Italy; francesco.triggiano@uniba.it (F.T.); francesca.apo@libero.it (F.A.); giusy.diella@uniba.it (G.D.); f.fasano@regione.puglia.it (F.F.); pasquale.stefanizzi@uniba.it (P.S.); marcolopuzzo@gmail.com (M.L.); carla.calia@uniba.it (C.C.); chrysovalentinos.pousis@uniba.it (C.P.); angelo.marzella@uniba.it (A.M.); giovanna.barbuti@uniba.it (G.B.); giuseppina.caggiano@uniba.it (G.C.); 3 National Research Council (CNR), Water Research Institute (IRSA), Via F. De Blasio, 5, 70132 Bari, Italy; silvia.brigida@ba.irsa.cnr.it; 4 Department of Environment and Health, Istituto Superiore di Sanità, 00161 Rome, Italy; giuseppina.larosa@iss.it (G.L.R.); luca.lucentini@iss.it (L.L.); 5 Department of Food Safety, Nutrition and Veterinary Public Health, Istituto Superiore di Sanità, 00161 Rome, Italy; elisabetta.suffredini@iss.it

**Keywords:** coronavirus, SARS-CoV-2, wastewater-based epidemiology, surveillance

## Abstract

As a complement to clinical disease surveillance, the monitoring of Severe Acute Respiratory Syndrome Coronavirus 2 (SARS-CoV-2) in wastewater can be used as an early warning system for impending epidemics. This study investigated the dynamics of SARS-CoV-2 in untreated wastewater with respect to the trend of coronavirus disease 2019 (COVID-19) prevalence in Southern Italy. A total of 210 wastewater samples were collected between May and November 2020 from 15 Apulian wastewater treatment plants (WWTP). The samples were concentrated in accordance with the standard of World Health Organization (WHO, Geneva, Switzerland) procedure for Poliovirus sewage surveillance, and molecular analysis was undertaken with real-time reverse-transcription quantitative PCR (RT-(q) PCR). Viral ribonucleic acid (RNA) was found in 12.4% (26/210) of the samples. The virus concentration in the positive samples ranged from 8.8 × 10^2^ to 6.5 × 10^4^ genome copies/L. The receiver operating characteristic (ROC) curve modeling showed that at least 11 cases/100,000 inhabitants would occur after a wastewater sample was found to be positive for SARS-CoV-2 (sensitivity = 80%, specificity = 80.9%). To our knowledge, this is the first study in Italy that has applied wastewater-based epidemiology to predict COVID-19 prevalence. Further studies regarding methods that include all variables (meteorological phenomena, characteristics of the WWTP, etc.) affecting this type of wastewater surveillance data would be useful to improve data interpretation.

## 1. Introduction

Several studies have investigated the presence of Severe Acute Respiratory Syndrome Coronavirus 2 (SARS-CoV-2) in wastewater [1,2,3,4,5] in the world. Viral shedding in stool can occur in 50% of symptomatic, asymptomatic, pre- and post-symptomatic patients with coronavirus disease 2019 (COVID-19). The shedding duration at a load of 10^2^ to 10^8^ ribonucleic acid (RNA) copies/g varies among patients, with an average of 14–21 days [6,7,8,9,10,11]. 

Some authors [12,13,14] have reported a high correlation between the detection of SARS-CoV-2 in wastewater and the number of COVID-19 cases in the catchment area served by wastewater treatment plants (WWTPs), suggesting that the monitoring of wastewater could be a useful tool for predicting trends in COVID-19 prevalence. This approach, known as wastewater-based epidemiology (WBE), could solve certain limitations in existing surveillance systems that have been highlighted during the current COVID-19 pandemic or during previous ones (e.g., asymptomatic carriers, timing of diagnosis) [15].

To date, wastewater monitoring has been implemented as a successful strategy to track other health-related chemical and biological biomarkers, for example, in relation to illicit drug consumption, pharmaceutical use/abuse, water pollution, and the occurrence of antimicrobial resistance genes [16,17,18,19,20]. 

In Italy, the first detection of SARS-CoV-2 in a sewage sample was documented in northern Italy in December 2019 [21], though the first autochthonous case of COVID-19 was recognized in February 2020. Italy has been among the most severely affected countries in the world, to the point that the Ministerial Decree issued on 11 March 2020 [22] limited the movement of people throughout the nation and implemented social, recreational, and cultural lockdown measures that were enforceable [23,24,25]. In Italy, after a decline in infections owing to the vaccination campaign that began in January 2021, as of 21 July 2021, the national weekly incidence increased (19/100,000 inhabitants), primarily because of the circulation of the Delta variant [26]. This trend is similar to that of other European countries. To date, although the correlation between the occurrence of SARS-CoV-2 in wastewater and the number of COVID-19 cases [4,12,13,14] has been demonstrated, few studies have been conducted regarding the application of WBE to predict COVID-19 prevalence due to the complexity and uncertainties associated with the process [27].

Here, we report the first detection of SARS-CoV-2 RNA in untreated wastewater samples in Southern Italy, collected from WWTPs in the Apulia region. The aims of this study were: (1) To investigate the dynamics of SARS-CoV-2 in untreated wastewater with respect to the trend of COVID-19 cases in the region; (2) To apply WBE to predict COVID-19 prevalence. 

## 2. Materials and Methods

### 2.1. Study Design

Apulia is a region of Southern Italy that hosts a population of approximately 4 million inhabitants distributed in six provinces: Bari (BA), Barletta-Andria-Trani (BT), Brindisi (BR), Foggia (FG), Lecce (LE), and Taranto (TA). Apulia covers approximately 20,000 km^2^ and extends for 834 km along the coast.

**Figure 1:**
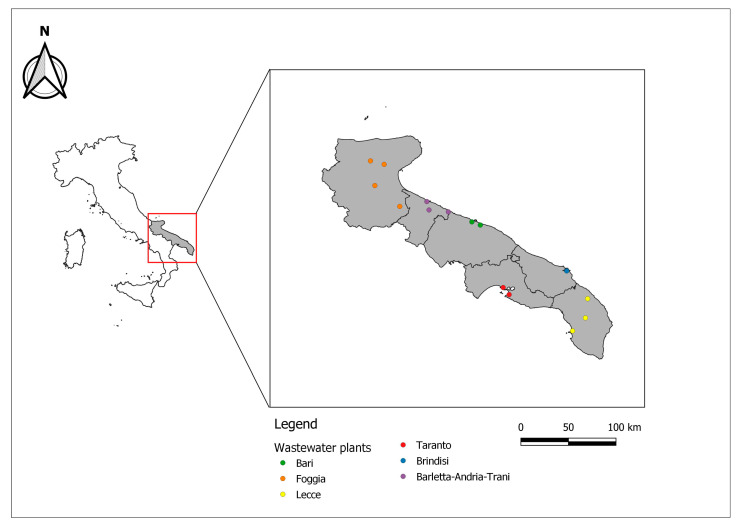
Locations of the included WWTPs in the Apulia region (Southern Italy).

The Apulian Water Agency manages the largest European aqueduct, with an approximate 20,000 km water network and a 10,000 km sewerage pipe network. Currently, 184 WWTPs (BA = 27; BT = 12; BR = 18; FG = 69; LE = 39; TA = 22) serve the region, covering 74% of total need [28]. Of these, 15 WWTPs were selected for the investigation and are uniformly distributed throughout the region: BA (two plants, A and B), BT (three plants, A–C), BR (one plant, A), FG (four plants, A–D), LE (three plants, A–C), and TA (two plants, A and B) (Figure 1). These WWTPs serve a total of 1,857,189 inhabitants (47.0% of Apulia’s population, Table 1).

### 2.2. Sample Collection

A total of 210 wastewater samples were collected from 15 Apulian WWTPs, as well as 28 from BA, 56 from FG, 42 from LE, 28 from TA, 42 from BT, and 14 from BR. The sampling of untreated wastewater was performed by the Regional Environmental Protection Agency twice per month from May to November 2020. Composite samples over a 24 h period were collected from the WWTP influent post the inlet screens, immediately stored at −20 °C, and dispatched frozen to a regional reference laboratory for SARS-CoV-2 analysis (the Environmental and Food Hygiene Laboratory of the Department of Biomedical Science and Human Oncology, University of Bari, Aldo Moro, Italy, hereafter referred to as EnLab). The samples were stored frozen until further analysis. The samples were processed using Class II biological safety cabinets, and standard precautions were applied (hand hygiene products and personal protective equipment such as gloves, gowns, and face and eye protection).

Before virus concentration, the samples were thawed and underwent a 30 min treatment at 57 °C to inactivate the possibly present infectious viral particles to increase the safety of the analytical protocol for both the laboratory personnel and the environment [2].

### 2.3. Virus Concentration

Sample concentration was carried out via a two-phase separation (the polyethylene glycol–dextran method) in accordance with the World Health Organization (WHO, Geneva, Switzerland) Guidelines for the Environmental Surveillance of Poliovirus [29], with modifications as reported by La Rosa et al. [2] to adapt the protocol to enveloped viruses. In brief, a wastewater sample (250 mL) was centrifuged to pellet the solids, and the pellet was stored at 4 °C for further processing. The clarified wastewater was mixed with dextran (22%), NaCl (5 N), and polyethylene glycol 6000 (29%), and the mixture was agitated on an orbital shaker for 30 min at 4 °C. The mixture was then transferred to a separation funnel and allowed to stand overnight at 4 °C. The bottom layer and the interphase were then collected drop-wise, and this concentrate was added to the pellet from the initial centrifugation. The combined sample was treated with chloroform (1: 5 *v*/*v*) and assayed for the presence of virus.

### 2.4. RNA Extraction

The concentrated sample underwent viral RNA extraction using the NucliSENS miniMAG semi-automated extraction system with magnetic silica, following the manufacturer’s instructions (bioMerieux, Marcy l’Etoile, France) with some modifications. In particular, the lysis phase was prolonged from 10 to 20 min, and a short centrifugation (2000× *g*, 1 min) was used to pellet the sediment. Additionally, instead of 50 μL, 100 μL of magnetic silica beads was added to each sample. The extracted nucleic acids were further purified by polymerase chain reaction (PCR) inhibitors using the OneStep PCR Inhibitor Removal Kit (Zymo Research, Irvine, CA, USA) and stored at −20 °C until molecular analysis.

After the RNA extraction, a portion of each sample was processed in the EnLab laboratory (Bari, Italy) for qualitative assessment, and a portion was transferred to the Istituto Superiore di Sanità laboratory (Rome, Italy, hereafter referred to as ISS) for quantitative assessment.

### 2.5. Real-Time Reverse-Transcription Quantitative PCR (Real-Time RT-(q) PCR) 

Real-time RT-(q) PCR analysis was performed as described in La Rosa et al. [19]. Briefly, each 25 μL reaction contained 5 μL of RNA, 12.5 μL of 2× reaction buffer provided with the AgPath-ID™ One-Step RT-PCR reagents (Applied Biosystems, Foster City, CA, USA, 1 μL of 25× RT-PCR enzyme mix, 1 μL of forward primer (12.5 μM), 1 μL of reverse primer (22.5 μM), 1 mL of probe (6.25 μM), 1.83 μL of nuclease-free water (not DEPC, diethylpyrocarbonate-treated), and 1.67 μL of detection enhancer for real-time PCR (Applied Biosystems, Foster City, CA, USA). The following primer and probe sequences were used: CoV-2-F: ACA TGG CTT TGA GTT GAC ATC T (code 2297); CoV-2-R: AGC AGT GGA AAA GCAT GTG G (code 2298); CoV-2-P: FAM-CAT AGA CAA CAG GTG CGC TC-MGBEQ (code 2299) [21]. The RT-PCR experiments were carried out in triplicate using the CFX96 Touch Deep Well Real-Time PCR System (Bio-Rad, Hercules, CA, USA) for detection and the Quant Studio 12K Flex (Applied Biosystems, Foster City, CA, USA) for quantification. Thermal cycling conditions included an initial reverse transcription step at 50 °C for 30 min, inactivation of reverse transcriptase at 95 °C for 10 min, and 45 cycles of amplification at 95 °C for 15 s and 60 °C for 45 s (30 s when using Quant Studio 12K Flex (Applied Biosystems, Foster City, CA, USA)) with the fast thermal profile). The cycle threshold (Ct) values of RT-qPCR were used as indicators of the copy number of SARS-CoV-2 RNA in the sewage samples, with lower Ct values corresponding to higher viral copy numbers. A Ct value less than 40 was interpreted as positive for SARS-CoV-2 RNA. The limit of detection (LOD_50_) and the limit of quantification (LOQ) for this assay were calculated in a previous study, as described in La Rosa et al., 2021 [21], and were found to be, on pure samples of target RNA, an LOD_50_ of 0.41 g.c./μL and an LOQ of 3.71 g.c./μL; in sewage samples, LOD_50_ and LOQ were 1.46 g.c./μL RNA and 7.35 g.c./μL, respectively. To construct a standard curve, the targeted region was synthetized and purified by BioFab Research (Rome, Italy) and quantified by fluorometric measurement (Qubit, Thermo Fisher Scientific, Waltham, MA, USA). Tenfold dilutions were used to construct the standard curve (range 5 × 10^0^–5 × 10^4^ copies/μL). In vitro-synthetized RNA containing the target region was used as an external amplification control to check for PCR inhibition.

### 2.6. Statistical Analyses

A descriptive analysis was performed with regard to the distribution of the results of the WWTP samples analyzed from each province.

To correlate the wastewater results to the number of COVID-19 cases during the May–November 2020 study period, we first correlated the number of cases to the number of patient specimen collection swabs taken per day in the area served by each WWTP.

This operation effectively reduced the underestimation of COVID-19 cases. The maximum daily number of swabs carried out in Apulia during the examined period was 10,265; thus, the following formula was applied in Equation (1):

A = No. of COVID-19 cases on day x in the area served by plant X × (10,265/No. swabs carried out on day x)(1)

where A represents the estimated number of cases on day x in order to eliminate the uncertainty owing to the number of swabs carried out on day x.

Subsequently, to report the estimated number of COVID-19 cases (A) in the population served by each plant, the following formula was applied in Equation (2):

B = (A/population served by plan X) × 100,000(2)

where B represents the number of COVID-19 cases/100,000 inhabitants served by each plant. This operation allowed us to compare the number of cases that occurred in areas with different population sizes.

After the preliminary operations, R software version 4.0.5 (Brandon Greenwell, Cincinnati, Ohio) was used for the statistical analysis, and a *p*-value < 0.05 was considered statistically significant.

Three types of analyses were carried out with regard to the occurrence of COVID-19 during the 15 days before and after wastewater sampling:

1. Chi-squared (χ^2^) test with Yates’s correction and the odds ratio to compare the percentage of COVID-19 cases in relation to the results of the wastewater sample for SARS-CoV-2 in the following time periods:

- 15 days before: positive vs. negative wastewater samples
- 15 days after: positive vs. negative wastewater samples
- 15 days before vs. 15 days after: positive wastewater samples
- 15 days before vs. 15 days after: negative wastewater samples

This analysis showed if and how many COVID-19 cases, detected previously and subsequently to wastewater samples positive for SARS-CoV-2, influenced the SARS-CoV-2 detection in wastewater.

2. A Poisson regression model was used to perform multivariate analysis on the viral load and the PCR Ct values of the positive wastewater samples in comparison with the following parameters:

- COVID-19 case trend in the 15 days before wastewater sampling
- COVID-19 case trend in the 15 days after wastewater sampling
- Population served by each plant
- Current average daily capacity (m^3^/d) of each WWTP

To standardize the different units of measurement of the four independent parameters, the data were normalized using the following Equation (3) [30]:

x normalized = (x−x min)/(x max−x min)(3)

where x is each of the four variables indicated above; x max: max value of each variable; x min: min value of each variable.

The final model included only variables with a *p*-value of <0.05 in the preliminary model of all variables. 

To quantify the effects of the above parameters on the viral load and Ct values, the relative risk (RR) of each parameter was calculated [31,32]. 

3. A receiver operating characteristic (ROC) curve was used to assess the trend of cases 15 days after each sampling event to identify an optimal cutoff value that could predict how many cases of COVID-19 per 100,000 inhabitants (served by the plant) might occur within 15 days after a positive wastewater sample. The ROC curve shows the tradeoff between the true positive fraction (TPF) and false positive fraction (FPF), and is generated by the plot of TPF (sensitivity) versus FPF (1-specificity) across varying cut-offs. The concept of an ROC curve is based on the notion of a "separator" (or decision) variable as one change in the criterion for positivity [33]. The ROC curve corresponding to the progressively greater discriminant capacity of diagnostic tests (max values of sensibility and specificity) are located progressively closer to the upper-left-hand corner in "ROC space". In our case, we calculated the cases that occurred 15 days after the wastewater sampling (both positive and negative for SARS-CoV-2). The ROC identified the optimal cut-off value, above which were included most of the positive wastewater samples for SARS-CoV-2 (sensitivity) and under which were included most of the negative wastewater samples (specificity).

## 3. Results

Overall, SARS-CoV-2 RNA was present in 12.4% (26/210) of the samples. In particular, 32.1% (9/28) of the wastewater samples from BA were positive, followed by 7.1% (4/56) from FG, 7.1% (3/42) from LE, 17.8% (5/28) from TA, and 11.9% (5/42) from BT. No positive samples were detected from BR.

The results reported by the EnLab laboratories were all confirmed by ISS, except in eight samples. The Ct and genome copies/liter (g.c./L) values of the positive samples are reported in Table 2. 

The virus concentrations of the positive samples ranged from 8.8 × 10^2^ to 6.5 × 10^4^ g.c./L. The highest concentrations were recorded in a sample from TA-B in November 2020 (6.5 × 10^4^ g.c./L) and in a sample from FG-C in May 2020 (6.2 × 10^4^ g.c./L). 

The distribution of the number of cases in the presence of positive wastewater samples in the study area is shown in Table 3.

Table 4 and Table 5 present the COVID-19 test swab results in relation to SARS-CoV-2 detection in wastewater from the Apulia region during the study period in the areas served by the investigated WWTPs. Table 4 shows the results from the 15 days before wastewater sampling, and Table 5 shows the results from the 15 days after sampling.

The χ^2^ results with Yates’s correction and odds ratios are reported in Table 6.

Compared with wastewater samples that are negative for SARS-CoV-2, when wastewater samples are positive, there is a 45.8-fold greater risk of cases in the 15 days prior to sampling and a 32.5-fold greater risk in the 15 days after sampling.

Compared with the results 15 days before wastewater sampling, when samples are positive for SARS-CoV-2, there is a two-fold greater risk of swabs also being positive for SARS-CoV-2 (and therefore a two-fold risk of identifying COVID-19 cases) in the 15 days after sampling.

Table 7 presents the Poisson regression modeling results to evaluate whether independent parameters affected the dependent variable "SARS-CoV-2 viral load" in wastewater samples (mean load = 1825.90 g.c./L, first and third interquartile = 0, median load = 0, range = 0–65,000).

**Figure 2:**
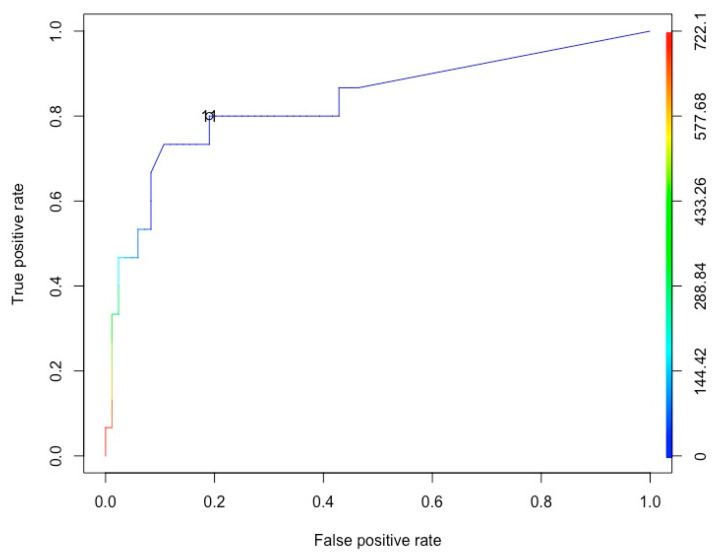
ROC curve to predict the number of COVID-19 cases/100,000 inhabitants 15 days after SARS-CoV-2 detection in wastewater (11cases/100,000 inhabitants).

All independent parameters had a significant influence on the wastewater SARS-CoV-2 detection results. In particular, the average daily capacity of the WWTPs was inversely proportional to the SARS-CoV-2 load of the wastewater (the lower the daily average capacity, the greater the probability of detecting the virus). By contrast, the other three parameters were directly proportional to the viral load. When the Ct value was used as a dependent variable in the Poisson regression, none of the four independent parameters listed in Table 7 had a statistically significant impact. To predict the number of COVID-19 cases/100,000 inhabitants served by the WWTPs in the 15 days after sampling, an ROC curve model was applied using the wastewater samples that were positive for SARS-CoV-2 (Figure 2). The analysis showed a cut off value for which at least 11 cases/100,000 inhabitants would occur after a wastewater sample was found to be positive for SARS-CoV-2 (sensitivity = 80%; specificity = 80.9%).

## 4. Discussion

The EU Commission recommendation of 17 March 2021 strongly encourages member states to establish national wastewater surveillance systems to detect SARS-CoV-2 and its variants in wastewater [35]. These systems should be implemented as soon as possible and no later than 1 October 2021.

Our study found that SARS-CoV-2 was detected in wastewater during the period in which cases were being diagnosed within the municipalities served by the investigated WWTPs. 

The virus circulation generally slowed during the summer months, as evidenced by the low incidence of cases during this time. The presence of SARS-CoV-2 in wastewater in July and September 2020 from the Bari (A+B) and Lecce (B) plants, respectively, which served municipalities without newly confirmed cases, could be related to undiagnosed asymptomatic cases and to previous cases, as the virus is excreted in the stool for some time after infection (approximately 30 days) [12]. After a small number of detections during the summer months, the presence of SARS-CoV-2 was again detected in all of the WWTPs, except for those in Brindisi. During this time, there was a concomitant increase in cases.

The positive samples were qualitatively and quantitatively confirmed by EnLab and ISS, respectively, and appeared to be related to a high level of the virus circulating in the population. The current detection methods are not sensitive enough to detect low quantities of viral RNA in wastewater because of the complex nature of this media [36]. 

The non-detection of virus by the ISS in some wastewater samples may be because of viral RNA degradation, owing to transportation conditions [13,37]. A recent study found that SARS-CoV-2 RNA is partially stable at 4 °C for at least 14 days [38]; however, one review stated that freezing and thawing the sample from −20 °C or −80 °C could lead to the degradation of the SARS-CoV-2 genetic material [39].

In line with previous studies [3,13], we found a direct correlation between the SARS-CoV-2 RNA concentration in wastewater and the number of COVID-19 cases during the 15 days before and after a positive detection in wastewater. The average daily capacity of the WWTPs was inversely proportional to the SARS-CoV-2 load in the wastewater samples, probably owing to dilution (e.g., precipitation, average daily water usage). 

To ensure that differences in viral concentration could not be attributed to changes in population, some authors have inserted an important step in the application of WBE, namely population normalization. For this purpose, human biomarkers as ammonium excreted in urine can be used to estimate the serviced population in an area via statistical modeling [15]. 

As has been demonstrated in previous examples such as the 2013–2014 silent polio epidemic in Israel [40], environmental surveillance can be used as a tool to decide when to enact restrictions, with the aim of an early introduction and avoiding premature repeal [41,42]. This surveillance approach could also be used to inform vaccination distribution [43] and to investigate emerging genomic variants circulating in the population [44,45,46]. 

The presence of SARS-CoV-2 in wastewater can be used to predict COVID-19 cases, supporting the potential of wastewater-based epidemiology (WBE) [1,8,21,47]. This approach represents a non-invasive early-warning tool for monitoring the status and trends of COVID-19 infection [48]. Here, we predicted that at least 11 cases/100,000 inhabitants would occur in the 15 days after detecting a positive wastewater sample. To our knowledge, this is the first study in Italy to use WBE to predict the COVID-19 prevalence. 

However, the usage of WBE for estimating COVID-19 prevalence remains limited, owing to the complexity and uncertainties associated with the process [37]. Several studies [28,38,49] have discussed the uncertainties in using WBE to assess SARS-CoV-2 prevalence. For viral shedding, variations in the magnitude, probability, and duration were commonly observed across different studies [27]. Physiological factors such as gender, age, and pathological conditions impact the probability of virus shedding among patients. For most viruses, the water matrix plays an important role in their inactivation and decay because, without active human cells as hosts in wastewater, the infectivity of SARS-CoV-2 was reported to be reduced [50]. From currently available reports, even for the best recovery method, a considerable loss of virus RNA is commonly observed [38]. The flow inside the sewers has relatively large uncertainties due to seasonal or diurnal variations in water usage patterns among the population and any rainfall event [51]. To date, the exploration for sampling techniques in the detection of SARS-CoV-2 RNA is limited, even if the use of the composite vs. grab sampling technique is preferable due to the inherent variability in virus shedding and diurnal sewer flows [37].

One limitation of our study is that it does not take into account several parameters that influence the result, such as the precipitation, catchment size, variation of the viral load in stool, virus degradation and dilution in the WWTP, the impact of the wastewater matrix components, and the underestimation of cases owing to asymptomatic patients [7,37,41]. Moreover, the duration and distribution of SARS-CoV-2 RNA shedding in feces varies among individuals and across time may be also affected by variants and vaccination [7,10,14,26].

The EU Commission recommendation [35] states that “wastewater surveillance is a tool to observe trends and not an absolute means to draw conclusions about the prevalence of COVID-19 in the population.” Therefore, further studies of the complex methods that include all variables that affect this type of wastewater surveillance data would be useful to improve data interpretation [28,37]. Moreover, a future development of our research (with a larger number of wastewater samples to make the analysis more robust) could foresee a validation of the statistical model used by comparing these results with those derived from innovative approaches such as an artificial neural network (e.g., machine learning) [27]. 

## 5. Conclusions

Wastewater surveillance is less resource-intensive than large-scale clinical testing, making it an optimal tool for long-term virus monitoring and for the early identification of viral circulation in a population. The early detection of SARS-CoV-2 RNA in wastewater could signal imminent danger, providing authorities with valuable time in which to coordinate and implement actions to slow disease spread. 

## Figures and Tables

**Table 1:** Population and current capacity of the included WWTPs (source: Apulian Water Agency, http://www.aqp.it, accessed on 1 August 2021 [28]).

Province and WWTP ^a^	Served population ^b^	Current capacity ^c^ (m^3^/d) ^d^
BA-A	224,830	84,854
BA-B	380,924	66,634
BT-A	130,000	13,907
BT-B	127,728	15,061
BT-C	66,232	12,960
BR-A	116,022	18,000
FG-A	183,695	33,148
FG-B	75,895	10,000
FG-C	15,969	2150
FG-D	33,789	4900
LE-A	43,302	6344
LE-B	37,576	10,546
LE-C	147,307	25,753
TA-A	34,754	8757
TA-B	239,166	34,045

^a^ WWTP: Wastewater treatment plant; ^b^ Population connected to the wastewater treatment plant; ^c^ Average water flow observed during the study period; ^d^ m^3^/d, water flow expressed as volume per day; BA-A, Bari-plant A; BA-B, Bari-plant B; BT-A, Barletta-Andria-Trani, plant A; BT-B, Barletta-Andria-Trani, plant B; BT-C, Barletta-Andria-Trani, plant C; BR-A, Brindisi, plant A; FG-A Foggia, plant A; FG-B, Foggia, plant B; FG-C, Foggia, plant C; FG-D, Foggia, plant D; LE-A, Lecce-plant A; LE-B, Lecce-plant B; TA-A, Taranto-plant A and TA-B, Taranto-plant B.

**Table 2:** Positive SARS-CoV-2 detections in wastewater samples by qualitative (Ct value) and quantitative (g.c./L) real-time PCR, May–November 2020.

Province and WWTP	Sampling Date (Year 2020)	EnLab	ISS
		Ct Value	g.c./L
BA-A	October 6	36.11	n.d.
	October 22	36.93	n.d.
	November 2	37.62	3.5 × 10^3^
	November 16	36.17	4.8 × 10^3^
BA-B	July 27	38.73	1.4 × 10^3^
	October 6	37.02	n.d.
	October 22	37.81	8.8 × 10^2^
	November 2	36.17	1.7 × 10^3^
	November 16	35.46	4.8 × 10^3^
BT-A	November 10	35.94	4.1 × 10^3^
	November 23	35.43	5.1 × 10^3^
BT-B	October 22	37.19	1.5 × 10^3^
	November 10	38.42	1.3 × 10^3^
	November 23	35.02	1.5 × 10^4^
FG-A	October 20	37.57	n.d.
FG-C	May 7	32.04	6.2 × 10^4^
	May 29	34.04	1.2 × 10^4^
	June 17	33.63	3.4 × 10^4^
LE-B	September 16	37.19	n.d.
	October 29	38.12	n.d.
	November 4	38.15	1.4 × 10^3^
TA-A	September 8	37.12	n.d.
	November 17	37.09	2.4 × 10^3^
TA-B	October 27	38.47	n.d.
	November 3	38.24	6.5 × 10^4^
	November 17	36.52	1.7 × 10^3^

g.c./L = genome copies/liter; n.d. = not detected; Ct = Cycle threshold; EnLab = Environmental and Food Hygiene Laboratory; ISS = Istituto Superiore di Sanità.

**Table 3:** Epidemiological data summary of COVID-19 cases in the study area and their relation to positive wastewater samples.

		30 April 2020		31 May 2020		30 June 2020		31 July 2020		31 August 2020		30 September 2020		31 October 2020		30 November 2020	
Province	WWTP	Cases ^a^	Incid ^b^	Cases ^a^	Incid ^b^	Cases ^a^	Incid ^b^	Cases ^a^	Incid ^b^	Cases^a^	Incid ^b^	Cases^a^	Incid ^b^	Cases ^a^	Incid ^b^	Cases ^a^	Incid ^b^
BA		1313	10.5	1483	11.8	1491	11.9	1504	12.0	1890	15.1	3034	24.2	7668	61.4	20,839	166.4
	A+B	19	0.3	27	0.4	28	0.4	28	0.4	40	0.6	68	1.1	2105	33.3	2844	47.02
BT		373	9.6	380	9.7	380	9.7	382	9.8	442	11.3	694	17.8	1989	51.0	6186	158.6
	A+B	2	0.1	2	0.1	2	0.1	2	0.1	2	0.1	2	0.1	578	22.3	1544	59.90
FG		1044	16.8	1155	18.2	1170	18.8	1186	19.1	1377	22.1	1887	30.3	4414	70.9	12,589	202.3
	A	10	0.54	4	0.21	3	0.16	0	0	6	0.32	7	0.38	50	2.72	873	47.5
	C	8	0.3	10	0.4	11	0.4	11	0.4	11	0.4	12	0.5	62	2.4	252	157.8
LE		487	6.1	515	6.5	521	6.6	557	7.0	670	8.4	797	10.0	1287	16.2	4119	51.8
	B	1	0.3	1	0.3	1	0.3	1	0.3	1	0.3	1	0.3	3	1.1	44	11.7
TA		258	4.5	281	4.9	281	4.9	281	4.9	313	5.4	540	9.4	1851	32.1	6408	111.1
	A+B	16	0.5	16	0.5	17	0.5	17	0.5	17	0.5	24	0.7	374	10.9	1316	48.04

^a^ Data from “Epidemia COVID-19—Bollettino Epidemiologico Regione Puglia” (http://www.regione.puglia.it/web/speciale-coronavirus/elenco-notizie, accessed on 1 August 2021) [34]; ^b^ Cumulative Incidence: the percentage of diagnosed cases per 10,000 inhabitants; Bold = cases concomitant with positive wastewater samples.

**Table 4:** Swab results in relation to SARS-CoV-2 detection in wastewater in the 15 days preceding wastewater sampling.

Outcome of Wastewater Samples for SARS-CoV-2	No. (%) of Negative Swabs for SARS-CoV-2	No. (%) of Positive Swabs for SARS-CoV-2
Negative	453,911 (99.9)	529 (0.1)
Positive	77,049 (95.0)	4101 (5.0)
Total	530,960 (99.1)	4630 (0.9)

**Table 5:** Swab results in relation to SARS-CoV-2 detection in wastewater in the 15 days following wastewater sampling.

Outcome of Wastewater Samples for SARS-CoV-2	No. (%) of Negative Swabs for SARS-CoV-2	No. (%) of Positive Swabs for SARS-CoV-2
Negative	453,163 (99.7)	1277 (0.3)
Positive	74,350 (91.6)	6800 (8.4)
Total	527,513 (98.5)	8077 (1.5)

**Table 6:** The percentage of COVID-19 cases in relation to the wastewater sample outcomes for SARS-CoV-2 detection in the 15 days before and after sampling.

Period Analyzed with Respect to Wastewater Sampling	Outcome of Wastewater Sample for SARS-CoV-2	Percentage of Cases with Respect to Wastewater Sample Outcome for SARS-CoV-2	χ^2^ with Yates’s Correction	Odds Ratio (95% CI)
15 days before	Positive vs. negative	5.0% vs. 0.1%	χ^2^ = 19.579 *p*-value < 0.0001	45.8 (41.7–50.0)
15 days after	Positive vs. negative	8.4% vs. 0.3%	χ^2^ = 30.398 *p*-value < 0.0001	32.5 (30.6–34.5)
15 days before vs. 15 days after	Positive vs. positive	5.0% vs. 8.4%	χ^2^= 715.84 *p*-value < 0.0001	1.7 (1.6–1.8)
15 days before vs. 15 days after	Negative vs. negative	0.1% vs. 0.3%	χ^2^ = 309.59 *p*-value < 0.0001	2.4 (2.2–2.7)

95% CI = 95% confidence interval.

**Table 7:** Poisson regression model of SARS-CoV-2 load in wastewater samples: final model.

	*β*	(eβ−1) = RR (%)	*p*-Value
Intercept	8.6647436		<0.0001 *
COVID-19 case trend in the 15 days after sampling	0.0067969	0.68	<0.0001 *
COVID-19 case trend in the 15 days before sampling	0.0640235	6.61	<0.0001 *
Daily average capacity (m^3^/d ^) of each WWTP	−0.3440558	−29.11	<0.0001 *
Population served by each plant	0.1569391	16.99	<0.0001 *

*β* = coefficient of the regression model for each independent variable; RR = Relative risk; * statistically significant; ^ d = day.
